# Monitoring of Polycyclic Aromatic Hydrocarbon Levels in Mussels (*Mytilus galloprovincialis*) from Aquaculture Farms in Central Macedonia Region, Greece, Using Gas Chromatography–Tandem Mass Spectrometry Method

**DOI:** 10.3390/molecules26195953

**Published:** 2021-09-30

**Authors:** Constantina Grigoriou, Danae Costopoulou, Irene Vassiliadou, Dimitrios Chrysafidis, Vassilios Tzamtzis, Evangelos Bakeas, Leondios Leondiadis

**Affiliations:** 1 Mass Spectrometry and Dioxin Analysis Laboratory, INRASTES, NCSR ‘‘Demokritos”, 15341 Athens, Greece; kgrigoriou@rrp.demokritos.gr (C.G.); costodan@rrp.demokritos.gr (D.C.); vasilirn@rrp.demokritos.gr (I.V.); 2 General Chemical State Laboratory–Food Division, 16 An. Tsocha, 11521 Athens, Greece; d.chrysafidis@gcsl.gr (D.C.); v.tzamtzis@gcsl.gr (V.T.); 3 Laboratory of Analytical Chemistry, Department of Chemistry, National and Kapodistrian University of Athens, Zografos, Panepistimiopolis, 15784 Athens, Greece; bakeas@chem.uoa.gr

**Keywords:** *Mytilus galloprovincialis*, PAHs, GC-MS/MS, North Aegean

## Abstract

A new sensitive and selective gas chromatography tandem mass spectrometry (GC-MS/MS) method was developed for the analysis of 26 polycyclic aromatic hydrocarbons (PAHs), including 16 Environmental Protection Agency (EPA) and 15 + 1 European Union (EU) PAHs, in mussel samples from aquaculture farms in Thermaikos and Strymonian Gulf, Central Macedonia Region, in three sampling periods. Concentrations were found at moderate to low values at all sampling sites, without exceeding maximum levels set by EU. Low molecular weight PAHs were predominant in all samples. Seasonal variation of the concentrations was observed; values were slightly higher in the winter period. Use of diagnostic ratios for potential sources of PAHs showed both petrogenic and pyrolitic origin. In comparison to other related studies of mussels from the Mediterranean Sea, Greek mussels cultivated in the studied gulfs are low in contaminants due to minimal environmental pollution effects. Low concentrations of PAHs are in compliance with the low values of other POPs which were found in the mussels.

## 1. Introduction

Polycyclic aromatic hydrocarbons (PAHs) are a large class of widespread organic compounds with two or more fused aromatic rings. They are produced as a result of incomplete combustion or pyrolysis of organic matter through both anthropogenic and natural processes. PAHs are non-polar and highly lipophilic compounds, which after their emission into the environment are widely distributed in the air and in particulate matter, water, soil and sediments [1]. They are considered Persistent Organic Pollutants (POPs) by the European Environment Agency of European Commission [2]. The Environmental Protection Agency has listed 16 compounds as priority pollutant PAHs [3]. The European Union (EU) recommends the monitoring of 15 + 1 PAHs which according to the Scientific Committee of Food (SCF) “show clear evidence of mutagenicity/genotoxicity in somatic cells in experimental animals in vivo and with the exception of benzo[g,h,i]perylene have also shown clear carcinogenic effects in various types of bioassays in experimental animals” [4].

Food is the main source of exposure to PAHs for a nonsmoker, while for smokers the contribution of cigarette smoke plays the major role [5,6]. The presence of PAHs in various foodstuffs is in mixtures and is attributed to environmental contamination, processing and storage conditions, and different cooking methods (grilling, roasting, and frying) [7]. PAHs in non-processed foods, such as vegetables and fruits, can result from the deposition of particulate matter on their waxy surface, or from the contamination of soil. Nevertheless, they are also considered to be produced by autogenous biosynthesis [8].

Their presence in fish and seafood depends on the ability of those organisms to metabolize them, since they are ubiquitous in the marine environment [9,10]. Mussels, clams, oysters and other filtrating organisms tend to bioaccumulate PAHs in their tissues as a result of their limited capacity to metabolize these contaminants [11,12], in contrast to fish, which have the ability to metabolize them through P-450 cytochrome oxidase. Hence, molluscs and particularly mussels are used as bioindicators of the contamination of various coastal areas and the marine environment from anthropogenic sources [13,14]. *Mytilus galloprovincialis*, the predominant species of bivalves in the Mediterranean Sea, is the main species recommended for performing biomonitoring studies [15]. 

The European Food Safety Authority (EFSA) Panel on Contaminants in the Food Chain (CONTAM Panel), based on the available toxicity data, covers the 15 + 1 PAHs in the opinion about PAHs in food, but considers that the risk characterisation should be based only upon available oral carcinogenicity data. Thus, apart from benzo[a]pyrene, which is not by itself considered a suitable indicator for the occurrence of PAHs, PAH4 (benzo[a]pyrene, benz(a)anthracene, benzo[b]fluoranthene and chrysene) and PAH8 (benzo[a]pyrene, benz(a)anthracene, benzo[b]fluoranthene, benzo[k]fluoranthene, chrysene, benzo[g,h,i]perylene, dibenz[a,h]anthracene and indeno(1,2,3-cd)pyrene) are considered the most suitable indicators of the presence of PAHs in food, even though PAH8 does not provide much additional information compared to PAH4. The European Commission has set maximum levels for benzo[a]pyrene and PAH4 in bivalve molluscs (fresh, chilled or frozen) at 5.0 μg kg^−1^ and 30.0 μg kg^−1^, respectively [16].

Regarding the presence of PAHs in bivalve molluscs from the Mediterranean Sea, previous studies have shown a wide range of concentrations depending on the contamination of the examined area. The mussel-transplantation technique in particular, indicates that in marine environments with pollution input from industrial and petroleum activities, refineries, urban emissions and discharges, and shipyard activities, PAH concentrations in molluscs are significantly higher compared to those of molluscs from unpolluted environments [17]. In order to assess possible sources, diagnostic ratios have been used in several studies [18]. In general, sources of PAHs in the Mediterranean Sea are numerous and difficult to determine. Gas chromatography–mass spectrometry (GC-MS) [19,20,21], High Resolution Gas Chromatography–High Resolution Mass Spectrometry (HRGC-HRMS) [22], High Performance Liquid Chromatography (HPLC) coupled with Ultraviolet/Visible Detector (UV/VIS) [23,24] or Fluorescence Detector (FLD) [25,26] are commonly the instruments used for the analysis of PAHs in mussels.

Greek mussels (*Mytilus galloprovincialis*) are an important export product. Studies in wild and cultivated mussels from various coastal areas of Greece, such as Elefsina, Salamina, Pagasitic and Thermaikos Gulf in the Aegean Sea, have shown that PAHs of low molecular weight are predominant, due to intense maritime activity, industrial and other anthropogenic activities [27]. So far there is no information available on PAH levels in farmed mussels from the Strymonian Gulf and limited information is available on farmed mussels from the Thermaikos Gulf, a site affected by various anthropogenic activities. These gulfs are considered the most important maritime areas in Greece for the culturing of farmed mussels, while large parts of these estuaries are located in wetland areas protected by the National–Community Legislation (Natura 2000, Ramsar Convention).

The aim of this study was the monitoring of 16 EPA, 15 + 1 EU PAHs, benzo[e]pyrene and perylene contamination of farmed mussels cultured in the Thermaikos and Strymonian Gulfs in the Central Macedonia Region, in order to assess the chemical contamination of the aquatic environment. A sufficiently sensitive and selective method, using isotope dilution and GC-MS/MS spectrometry, was developed, validated and implemented to detect PAHs and pinpoint possible contamination, even in trace levels. PAH concentration data could be further combined with information on the levels of other POPs, such as polychlorinated biphenyls (PCBs), polychlorinated dibenzo-p-dioxins and dibenzofurans (PCDD/Fs), in mussels from the area, for the evaluation of synergistic effects and the potential human health risk from mussel consumption.

## 2. Results and Discussion

### 2.1. Extraction

Prior to analysis of the samples, all materials and reagents were checked for the presence of PAHs. The choice of the extraction of PAHs using liquid–liquid partition with dimethyl sulfoxide (DMSO) was based on various tests that were performed with a number of extraction techniques, such as Soxhlet extraction and sonication. Hexane and mixtures of hexane/dichloromethane were used for the extraction of PAH from mussel tissue, as they are the most common solvents used for this purpose in a number of studies. Nevertheless, other interfering organic compounds were extracted along with PAHs leading to significant levels of chromatographic noise. Marine and freshwater mussels are filter feeders; they feed on plankton and other microscopic sea creatures, which contain chlorophyll in their cells. Hexane and hexane/dichloromethane mixtures dissolved the chlorophyll, which could not be retained afterwards in a silica clean up stationary phase. Thus, in order to avoid an alkaline treatment that could lead to low recovery values, liquid–liquid partition with DMSO was tested with satisfying results and acceptable recovery values. The extraction scheme with DMSO liquid–liquid partition was based on those described in previous studies [28,29].

### 2.2. Clean-Up

Among the various clean up procedures that are described for purification of the extracts, column chromatography with silica gel or basic alumina as a stationary phase was preferred due to its capacity to retain fat and other interferences [30]. The parameters of the clean-up stage, types and quantities of the stationary phases and ratios of the elution solvents were optimized. Basic and acid silica, treated with NaOH and H_2_SO_4_ respectively, were tested; however, the strong alkaline and acidic conditions had a decomposing effect, especially on low molecular weight PAHs, so neutral silica was preferred. Furthermore, evaporation until dryness was avoided in all steps due to the possible loss of low molecular weight PAHs, such as naphthalene and acenaphthylene [31].

### 2.3. Optimization of Mass Spectrometry and Chromatography Parameters

Due to the high stability of PAHs, the use of MS/MS was found suitable for the required enhanced selectivity and sensitivity. After the selection of precursor ion, different energy voltages were applied to the collision cell resulting in two monitored transitions for every PAH. Concerning the chromatographic separation, the performance of two different stationary phases for GC was evaluated; a typical 5% phenyl- 95% methylpolysiloxane substitution (DB-5MS) and a Select PAH column, designed specifically for PAH analysis. Separation of some isomers with the DB-5MS column was impossible, even though different column temperature programs and injector temperatures were applied. Chrysene could not be separated from triphenylene and benz(a)anthracene, cyclopenta[c,d]pyrene was partially separated from benz(a)anthracene, while benzo[b]fluoranthene, benzo[j]fluoranthene and benzo[k]fluoranthene were co-eluted, in all cases. The Select PAH column was clearly more selective, allowing the separation of all former crucial isomeric groups, giving chromatograms with good peak separation and better signal of the heavier PAHs dibenzo[a,e]pyrene, dibenzo[a,h]pyrene, dibenzo[a,i]pyrene and dibenzo[a,l]pyrene.

### 2.4. Validation Parameters

The method was validated in order to fulfill the performance criteria set by the European Commission Regulation 836/2011[32]. The quantification of concentrations and recovery values was carried out by the isotopic dilution method. Deuterated PAHs were used as internal standards. The recoveries ranged between 62–119%. Relative response factors for each compound were constant (Coefficient Variation < 20%) over a five-point calibration range. Limits of detection (LOD) ranged between 0.006 and 0.033 μg kg^−1^, while limits of quantitation (LOQ) ranged between 0.02 and 0.10 μg kg^−1^. The values of LODs and LOQs were significantly lower than those required by 836/2011 EC for each of the PAH4, 0.3 and 0.9 μg kg^−1^, respectively. According to literature, LOD values in PAH analysis of mussels range between 0.01 and 0.960 μg kg^−1^ w.w. [9,33,34,35] and from 0.050 to 10 μg kg^−1^ d.w. [19,36,37,38]. Based on these values, our method is considered sensitive enough and suitable for monitoring PAHs in background levels for risk assessment purposes. Trueness and precision were tested by replicate analysis of spiked samples. Trueness values ranged between −0.67% and +18.67 and RSD < 20%. Data regarding analytical validation parameters are available in Supplementary material Table S1. The average concentration values and precision values (RSD) calculated for each of the PAH4 of EC 836/2011 and their sum are shown in Table 1.

The accuracy and precision of the method was confirmed by the analysis and quantification of proficiency test samples for interlaboratory studies organized by the European Union Reference Laboratory (EURL) for Polycyclic Aromatic Hydrocarbons. z-scores were calculated and found within the range of satisfactory performance. Results and mass spectra are shown in Supplementary material Table S2 and Figures S1 and S2 respectively.

### 2.5. Levels of PAHs in Mussels

#### 2.5.1. Concentration Levels

The concentrations of 26 individual PAHs were determined in 51 mussel samples from three sampling areas (Supplementary material Table S3). The S1 area, consisting of nine sampling stations, is located in Thermaikos Gulf, 35 km from the city of Thessaloniki, where urban, industrial and shipping wastes from the city and itsport are discarded. The S2 area, consisting of five sampling stations, is also located in Thermaikos Gulf, but 60 km away from Thessaloniki in the Regional Units of Imathia and Pieria. The S3 area has three sampling stations in the Strymonian Gulf Regional Unit of Serres and Chalkidiki, a rural area without significant urban and industrial activity. Sampling was conducted in three sampling periods (spring, winter, summer) from the same stations. Sampling locations and number of farms are reported in Table 2.

PAHs were detected in all 51 mussel samples. Fluorene, fluoranthene, pyrene and phenanthrene were the dominating PAHs and were found in all samples. On the other hand, values of BghiP, DBahA, DBaiP, DBaeP, DBalP and DBahP were found below the LOQ and LOD in all samples. From the rest of the 26 PAHs determined, phenanthrene had the highest concentration levels, while indeno(1,2,3-cd)pyrene the lowest.

In all Σ_PAHs_ values, the upper bound values were used as the worst case scenario, even though there might be a slight overestimation in the total sums. Nevertheless, due to the low values of the limit of detection (LOD) and limit of quantitation (LOQ), the difference between lower and upper bound values was not significant. Upper bound concentrations are calculated on the assumption that all the values below the LOQ are replaced by the LOQ, while for lower bound concentrations all the values below the LOQ are replaced by zero [6]. LOD, LOQ, upper bound mean, total spatial mean and median concentrations, range of values for each individual PAH and Σ_PAHs_ per sampling site, are reported in Table 3.

Upper bound mean concentrations of BaP and Σ_PAHs_ per sampling period, total seasonal mean, and range of values per season are reported in Table 4. Results are expressed as μg kg^−1^ wet weight (w.w.).

The concentrations of Σ_26PAHs_ in the mussels ranged from 1.37 to 25.59 μg kg^−1^, with the lowest values corresponding to mussels sampled in the spring period from station S2 and the maximum values to mussels sampled from the S3 station in winter. The mean upper bound Σ_26PAHs_ for station S1, for all three sampling periods, was 5.96 μg kg^−1^, for S2 4.27 μg kg^−1^, and for S3 6.21 μg kg^−1^. Similarly, no major differences were observed in the total spatial Σ_16PAHs_, Σ_15+1PAHs_ and Σ_4PAHs_ means between the three sites. For the Σ_16PAHs_, mean values were 4.98, 3.21 and 4.88 μg kg^−1^ for sites S1, S2 and S3, respectively, while range of concentrations was between 1.07 and 15.36 μg kg^−1^ at S1, 0.76 and 11.18 at S2 and 1.25 to 19.42 at S3. Concerning the Σ_15+1PAHs_, mean values were 1.29, 1.42 and 2.32 μg kg^−1^ for sites S1, S2 and S3, respectively, and the range of concentrations was between 0.70 and 3.75 μg kg^−1^ at S1, 0.72 and 4.16 at S2 and 0.71 to 11.47 at S3. The spatial means for the Σ_4PAHs_ were 0.45 μg kg^−1^ for S1, 0.53 μg kg^−1^ for S2 and 0.98 μg kg^−1^ for S3. Values of the concentrations for the sum of 4 PAHs ranged between 0.08 and 2.42 μg kg^−1^ for mussels from S1, 0.09 and 2.35 μg kg^−1^ for mussels from S2 and 0.09 and 5.77 μg kg^−1^ for samples from S3. BaP was found at 0.09 μg kg^−1^ in mussels from S1 and S2 and at 0.29 μg kg^−1^ in those from S3, while the range of concentrations had a minimum < LOQ at all sampling sites, and a maximum of 0.47, 0.34 and 2.11 μg kg^−1^ at S1, S2 and S3, respectively.

**Figure 1:**
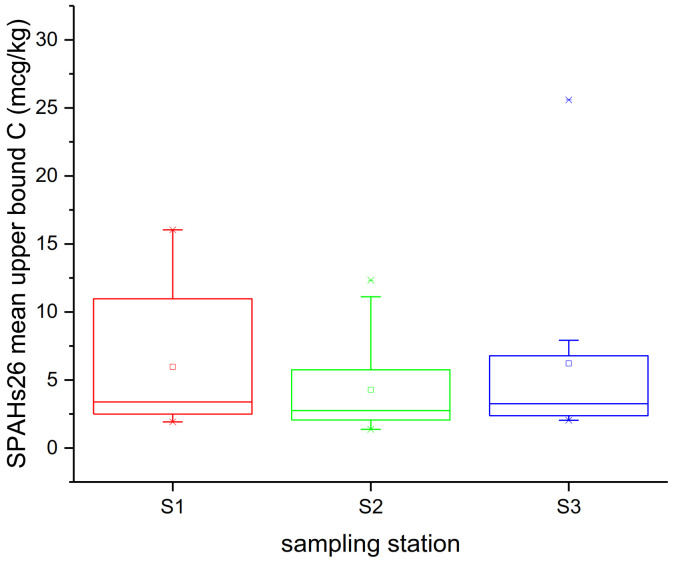
Spatial variation of mussels’ PAH contamination.

According to these levels, even though mussels from the S1 stations in the Thermaikos Gulf were expected to be more contaminated due to the industrial, shipping and anthropogenic activity from the city of Thessaloniki, no significant variation in the total mean concentrations of Σ_26PAHs_ was observed between the sites. A one-way ANOVA test did not determine significant differences among different sampling sites (*p* > 0.05) (Figure 1). Nevertheless, the range of the concentration values from the S1 samples, which was two times higher compared to S2 and S3, implies the presence of the aforementioned activity.

**Figure 2:**
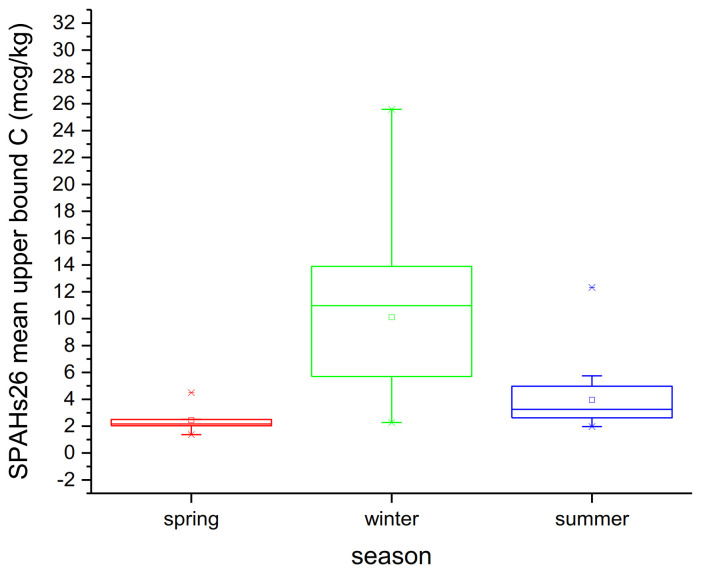
Seasonal variation of mussels’ PAH contamination.

On the other hand, an increase in PAH content observed during the winter sampling period, in all sampling sites, shows a seasonal contamination variation. Samples from the winter period had higher concentration levels for the sum of 26 PAHs, with a seasonal mean upper bound at 10.12 μg kg^−1^, followed by those from the summer sampling period with a Σ_26PAHs_ at 3.95μg kg^−1^; the lowest concentrations were determined in samples from the spring sampling period at 2.45 μg kg^−1^. Following the same seasonal distribution trend, the highest mean values for Σ_16PAHs_, Σ_15+1PAHs_ and Σ_4PAHs_ were determined in the winter period, followed by those in the summer period and finally those in the spring period. For BaP, the total range of concentrations between seasons varied from values < LOQ, as minimum and maximum values were 0.06 in spring, 2.11 in winter and 0.34 μg kg^−1^ in summer. Significant seasonal variation was observed for all three sites (*p* < 0.05) (Figure 2).

This result is in agreement with the literature and is attributed to different physiological conditions in the mussel populations, related to different stages in their lifecycle [19,21] and seasonal differences in biotic and abiotic factors regulating PAH metabolic mechanisms [24].

Samples were also analyzed at the General Chemical State Laboratory–A Chemical Service of Athens–Departement B, which is the National Reference Laboratory for PAHs in Food. The method used is based on ISO 15753:2006, using benzo[b]chrysene as internal standard and an HPLC/DAD/FLD Agilent 1100 Series system equipped with a Vydac 201 TP 54 column, as described previously in Costopoulou et al. [39]. Results showed higher individual concentrations for the light molecular PAHs such as phenanthrene and fluoaranthene, probably due to noise levels of the HPLC. LOD and LOQ levels were also higher, except for those of DBahA. None of the samples was found to exceed the maximum levels for the sum of four PAHs set by the E.U., which is 30.0 μg kg^−1^ for fresh, chilled or frozen bivalve molluscs. The established maximum level for BaP is 5.0 μg kg^−1^, while in the present samples the highest concentration level was 2.11 μg kg^−1^, in winter mussels originating from station S3.

According to the Scientific Opinion of the EFSA’s Panel on Contaminants in the Food Chain, the mean upper bound concentration in 187 fresh bivalve molluscs from several European countries was 1.36 μg kg^−1^ for BaP and 10.75 μg kg^−1^ for 4PAH (benzo[a]pyrene, chrysene, benz[a]anthracene and benzo[b]fluoranthene) [6]. In our study, the corresponding values were significantly lower; BaP mean values were 0.09 μg kg^−1^ in mussels from the S1 and S2 sites and 0.29 μg kg^−1^ for the samples from the S3 site, while values for the sum of four PAH were 0.45 μg kg^−1^ for S1, 0.53 μg kg^−1^ for S2 and 0.98 μg kg^−1^ for S3.

Taking into consideration that Mediterranean mussels have a moisture content of around 85% [40], the results of our study expressed as μg kg^−1^ d.w. have a range for the sum of 26 PAHs between 20.55 and 383 μg kg^−1^ d.w. (mean upper bound), for the sum of 16 EPA PAHs between 1.95 and 171.3 μg kg^−1^ d.w., for EU 15 + 1 PAHs between 10.5 and 172.05 μg kg^−1^ d.w. and for four PAHs values are from 1.2 to 86.55 μg kg^−1^ d.w. Kasiotis et al. have analyzed samples from the Saronic and Pagasitic gulfs as well as various coastal areas of Turkey with a GC-MS/MS method. The range of concentrations for Greek samples for the sum of 16 EPA PAHs was found between 5.7 and 518.1 μg kg^−1^ d.w. [41]. In another study, mussels cultivated in the Saronic, Pagasitic, and Corinthian Gulfs and islands have been analyzed with GC-MS technique and found to have a total concentration of 24 PAHs from 25 to 75 μg kg^−1^ d.w. [42]. Previously published data from the analysis of mussels, surface water and sediments from Elefsina, Salamina Gulf and Saronic Gulf using HPLC-FLD have shown a sum of concentrations of 16 PAHs in the mantles and gills of the mussels between 184 and 2453 μg kg^−1^ d.w. [27]. Levels of PAHs in mussels from the Thermaikos and Strymonian Gulf are lower than those reported for mussels from the Saronic Gulf, an area with intense maritime and industrial activity, as well as those from the Pagasitic Gulf.

In general, the values of PAHs determined in the present study are similar or lower than those found in wild or cultivated mussels from other Mediterranean countries. Previously reported mean values or ranges of mean values for the sum of 16 EPA PAHs were 22.4 μg kg^−1^ w.w. for mussels from Catalonia, Spain [22], 22–106 μg kg^−1^ d.w. for mussels from the North West Basin of the Mediterranean Sea [17] and for wild and commercial mussels from both Spain and Portugal values were 52.91 and 37.58 μg kg^−1^ w.w., respectively [18]. Additionally, high concentration levels for those PAHs were found in other studies, with the mean values for Σ_16PAHs_ ranging from 107.4 to 430.7 μg kg^−1^ d.w. in mussels from Tunisia and between 627–1550 μg kg^−1^ w.w. in mussels from Italy [21,25]. Thus, even though the results in other studies are usually expressed as lower bound and ours are expressed as upper bounds, and have a higher mean minimum value in some cases, maximum values are far lower and the range of concentrations is narrower than that reported in the literature.

The lower levels are attributed not only to the limited contamination of the examined areas compared to others, but also to the increased sensitivity of the MS/MS method compared to those such as the HPLC or single MS methods that are commonly used. These techniques may lead to incorrect calculations due to background noise, especially for Low Molecular Weight PAHs. Therefore, the selection of the MS/MS technique along with the analysis method was found suitable for the detection and quantification of PAHs in background levels, especially for risk assessment purposes in the field of environmental chemicals.

#### 2.5.2. Distribution Patterns and Diagnostic Ratios for PAH Origin

**Figure 3:**
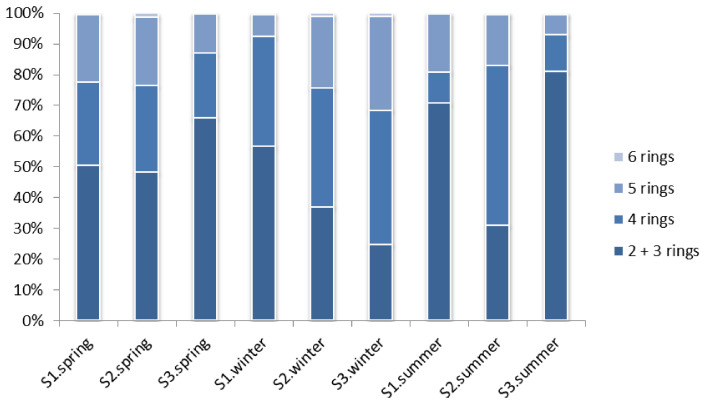
Distribution of PAHs in each sampling site and period according to number of rings.

The low molecular weight (LMW) PAH fraction is composed of compounds with two or three aromatic rings, while high molecular weight (HMW) PAHs contain four, five and six rings. To avoid misleading results in the distribution patterns and ratios caused by the different LOD and LOQ values among the compounds, all values below LOQ were replaced by zero (lower bound). As shown in Figure 3, LMW PAHs were predominant in the mussels collected from site S1 in all three sampling periods. For mussels coming from S2, LMW levels were higher in spring, but HMW, specifically 4-ring PAHs, were more abundant in the winter and summer periods. At S3, LMW compounds prevailed during spring and summer, but 4-ring PAHs contributed more to the winter contamination profile. Prevalence of LMW PAHs indicates petrogenic origin from crude oil and petroleum spillages, shipping and shipyard activities, industrial and vehicle emissions and urban discharges. PAHs with 4 aromatic rings could be from petrogenic sources but more likely from pyrolitic inputs, as the other HMW PAHs with five rings or more. These PAHs originate from various combustion processes of wood, coal etc. [43].

Diagnostic ratios have been proposed for the more accurate assessment of PAHs origin, such as LMW/HMW (>1 petrogenic origin), Phe/An (>15 petrogenic or <10 pyrolytic origin), Fa/Py (>1 pyrolitic origin) and Chr/BaA (<1 pyrolitic origin) [17,27]. The use of these ratios is more accurate in sediments and surface water, but is commonly applied in mussel samples as well. In our study, samples from S1 had ratios of LMW/HMW > 1, Phe/An ratio > 15 (except for Phe/An = 11.13 in the summer period) and Fa/Py > 1, in all sampling periods, clearly suggesting a petrogenic origin from the city of Thessaloniki, as expected. For mussels from stations S2 and S3, which are located in rural agricultural areas, seasonal variations among the diagnostic ratios were observed. According to the LMW/HMW and Phe/An ratio, petrogenic origin of PAHs was observed for samples in the S2 area in the spring period, while the corresponding values of those ratios for the winter and summer periods as well as the Fa/Py ratio demonstrate pyrolitic origin. Contamination of samples at the S3 stations was attributed to both petrogenic and pyrolitic processes, due to the differences in diagnostic ratios. At all three sites, the value of Chr/BaA ratio was in the range of petrogenic origin, nevertheless 4-ring PAHs may be attributed to by both petrogenic and pyrolitic sources.

The accurate determination of PAH sources is difficult for the S2 and S3 areas, since they are influenced by various sources depending on the season and anthropogenic activities. Overall, the S1 sampling site is clearly affected by petrogenic inputs in all three seasons, but the distribution pattern profiles of samples from the S2 and S3 sites seem to be affected by both pyrolitic and petrogenic sources.

In accordance with low PAH concentrations, unpublished results for PCDD/Fs, dioxin like-PCBs and non-dioxin like-PCB compounds in the same samples showed either undetectable or low concentration levels, with distribution patterns depending mostly on the season of sampling and not as much on the sampling area as was the case for PAHs.

## 3. Materials and Methods

### 3.1. Collection of Mussels

Samples of cultivated mussels (*Mytilus galloprovincialis*) were collected from aquaculture farms located in the Central Macedonia Region, Greece. Sampling farms were distributed in three different areas, two of them in the Thermaikos Gulf and one in the Strymonian Gulf. Mussels from nine sampling stations in the Thessaloniki Regional Unit were denoted as S1, from five stations in the Imathia and Pieria Regional Unit as S2 and from three sampling stations from Chalkidiki and Serres Regional Unit as S3. Sampling was conducted by the Department of Animal Health and Welfare, Veterinary Drugs and Application of the General Directorate of Agricultural Economy and Veterinary Medicine of the Central Macedonia Region, Greece, during three sampling periods: spring period in May 2018, winter period from February until May 2019 and summer period in July 2019 (Table 2). All samples were sent for PAH, PCDD/F and PCB analysis to the Mass Spectrometry and Dioxin Analysis Laboratory, National Centre for Scientific Research (NCSR), “Demokritos”, which is the National Reference Laboratory for Halogenated POPs in Feed and Food. Samples were delivered fresh inside an ice-cooled box in polyethylene packages. Each of the 51 samples consisted of 1.5 or 3 kg fresh mussels, which were cleaned and the flesh was removed, homogenized, and stored in glass bottles at −18 °C. Τhe age of the mussels was between 6 and 7 months when the desired commercial size of 5 cm was acquired.

### 3.2. Materials and Reagents

The compounds analyzed in the present study are shown in Table 5. PAH surrogate standard mixture of D_8_, D_10_, D_12_ and D_14_-labeled PAHs for the preparation of D-labeled internal standard solution was purchased from Cambridge Isotope Laboratories (Andover, MA, USA). Standard solution of ^13^C_12_-PCB 80 was purchased from Wellington Laboratories (Guelph, ON, Canada). Hexane and dichloromethane were purchased from Promochem (Wesel, Germany). Acetone, dimethyl sulfoxide (DMSO), sodium chloride, sodium oxalate, sodium sulfate and silica gel (60–200 mesh) were purchased from Merck (Darmstadt, Germany) and Basic Alumina activity Super 1 for dioxin analysis from MP Biochemicals (Eschwege, Germany). Traces of low molecular PAHs were found in silica gel and sodium sulfate, so thermal decomposition in an oven at 650 °C overnight was chosen. Afterwards, silica gel and basic alumina were activated in an oven at 200 °C overnight.

### 3.3. Preparation of Samples

#### 3.3.1. Extraction of PAHs

Ten grams of each sample were weighed in a 50 mL polypropylene centrifuge tube and spiked with 100 μL of a mixture of D-labeled PAHs 100 ng mL^−1^ before extraction. Five g sodium oxalate and 5 g sodium sulfate were added, then the samples were extracted with 30 mL hexane and homogenized with an Ultra-Turrax high-performance dispersing instrument. The mixture was vortexed for 1 min and then centrifuged at 5000 rpm for 10 min. The supernatant was obtained, evaporated in a rotary evaporator at 30 °C until 5 mL and transferred to a polypropylene centrifuge tube for liquid–liquid partition with 5, 3 and 2 mL DMSO in three stages. In each stage the mixture was vortexed and then centrifuged for 5 min. The hexane fraction was discarded while the DMSO extracts were combined (10 mL), followed by the addition of 24 mL of distilled water and 1.2 g of sodium chloride. For the back-extraction of PAHs 20, 10 and 6 mL of hexane were used in three stages, in which the mixtures were vortexed and then centrifuged for 5 min. The combined hexane extracts were evaporated in a rotary evaporator at 30 °C until 5 mL.

#### 3.3.2. Clean Up

The mixture was brought onto a column (30 cm length, 8 mm ID) plugged with glass wool and filled with 1 g silica gel, 5 g basic alumina, 4 g silica gel and 1 g of sodium sulfate. The column was rinsed twice with 2.5 mL hexane and then eluted with 100 mL of hexane/dichloromethane mixture (30:70 *v*/*v*). The fractions were collected, pre-concentrated with a rotary evaporator to 0.5 mL and transferred to glass tubes, where under a gentle stream of nitrogen they were evaporated till 100 μL. Finally, 20 μL of n-nonane containing 500 ng mL^−1^ of injection standard ^13^C_12_-PCB 80 were added and the liquid was transferred to injection vials.

### 3.4. Instrumental Analysis

The quantification of PAHs was performed on a Thermo TSQ Quantum XLS Ultra triple-quadrupole GC-MS/MS coupled to a Trace GC Ultra gas chromatograph equipped with a TriPlus autosampler (Thermo, North Kingstown, RI, USA) and an Agilent J&W Select PAH column 30 m, 0.25 mm, 0.15 µm (Agilent, Santa Clara, CA, USA). The sample solutions were injected in splitless mode and the injected volume was 2 μL. Helium was used as a carrier gas. The temperature of the injector was 260 °C and that of the transfer line 290 °C. The column temperature was programmed as follows: initial level 70 °C, held for 0.7 min, ramp to 180 °C at 85 °C/min, ramp to 230 °C at 3 °C/min, held for 7 min, ramp to 280 °C at 28 °C/min, held for 10 min, ramp to 350 °C at 14 °C/min and held for 3 min. For the optimization of the selected reaction monitoring method, different electron energy, emission current, source temperature, column temperature programs, collision energy, scan width and scan time values were tested. One precursor ion for each PAH resulted in two product ions that were measured and the ion abundance ratio between the two monitored product ions was checked with the same ratio of calibration standard at a similar concentration (Table 5).

**Table 5:** Mass transitions, retention time and collision cell energy for target compounds.

Compound	Abbrev.	Rt	Precursor Ion	Quantifier Ion (*m*/*z*)	Qualifier Ion (*m*/*z*)	Collision Cell Energy (eV)
Naphthalene	Na	2.75	127.89	128.00	102.00	15
Acenaphthylene	Acl	3.98	151.88	152.00	126.00	22
Acenaphthene	Ac	4.12	152.89	153.00	126.90	10
Fluorene	Fl	4.91	164.88	165.00	163.00	10
Phenanthrene	Phe	7.82	177.90	178.00	152.00	22
Anthracene ^a^	An	7.97	177.90	178.00	152.00	22
Fluoranthene	Fa	13.72	201.90	202.00	200.00	10
Pyrene	Py	15.37	201.90	202.00	200.00	10
Benzo[c]fluorene ^b^	BcFl	17.88	215.94	215.00	189.00	30
Benz(a)anthracene	BaA	25.61	227.93	226.00	202.00	22
Chrysene	Chr	26.30	227.90	226.00	202.00	22
Cyclopenta[c,d]pyrene ^c^	Cpp	25.99	225.90	226.00	224.00	22
5-methylchrysene ^c^	5MeChr	28.19	241.93	242.00	240.00	10
Benzo[b]fluoranthene	BbFa	30.91	251.90	252.00	226.00	22
Benzo[k]fluoranthene ^d^	BkFa	31.04	251.90	252.00	226.00	22
Benzo[j]fluoranthene ^d^	BjFa	31.12	251.90	252.00	226.00	22
Benzo[e]pyrene	BeP	32.85	251.90	252.00	226.00	22
Benzo[a]pyrene	BaP	33.20	251.90	252.00	226.00	22
Perylene	Per	33.99	251.90	252.00	226.00	22
Indeno(1,2,3-c d)pyrene	IP	40.17	275.86	276.00	274.00	22
Dibenzo[a,h]anthracene	DBahA	40.21	277.88	278.00	252.00	22
Benzo[g,h,i]perylene	BghiP	41.19	275.87	276.00	274.00	22
Dibenzo[a,l]pyrene ^e^	DBalP	43.90	301.85	302.00	300.00	5
Dibenzo[a,e]pyrene ^e^	DBaeP	44.84	301.85	302.00	300.00	5
Dibenzo[a,i]pyrene ^e^	DBaiP	45.39	301.85	302.00	300.00	5
Dibenzo[a,h]pyrene ^e^	DBahP	45.67	301.85	302.00	300.00	5
Naphthalene (D8)	Na (D8)	2.73	135.96	136.00	107.96	22
Acenaphthylene (D8)	Acl (D8)	3.93	159.93	160.00	131.97	22
Acenaphthene (D10)	Ac (D10)	4.07	163.96	164.00	162.00	22
Fluorene (D10)	Fl (D10)	4.86	175.96	176.00	174.00	10
Phenanthrene (D10)	Phe (D10)	7.71	187.97	188.00	159.99	22
Fluoranthene (D10)	Fa (D10)	13.6	211.85	210.05	208.04	10
Pyrene (D10)	Py (D10)	15.24	211.99	210.07	208.03	22
Benzo(a)anthracene (D12)	BaA (D12)	25.29	240.00	236.00	212.00	22
Chrysene (D12)	Chr (D12)	26.05	240.00	236.00	212.00	22
Benzo(b)fluoranthene (D12)	BbFa (D12)	30.77	263.96	260.00	236.00	35
Benzo(k)fluoranthene (D12)	BkFa (D12)	30.92	263.96	260.00	236.00	35
Benzo[a]pyrene (D12)	BaP (D12)	33.03	263.96	260.00	236.00	35
Perylene (D12)	Per (D12)	33.79	263.96	260.00	236.00	35
Indeno(1,2,3-cd)pyrene (D12)	IP (D12)	40.06	287.93	288.07	284.00	35
Dibenz[a,h]anthracene (D14)	DBahA (D14)	40.08	291.95	292.00	288.00	35
Benzo[g,h,i]perylene (D12)	BghiP (D12)	41.1	287.93	288.07	284.00	35

^a^ Phenanthrene (D10) is used as internal standard; ^b^ Pyrene (D10) ) is used as internal standard; ^c^ Chrysene (D12) is used as internal standard.; ^d^ Benzo(b)fluoranthene (D12) is used as internal standard. ^e^ Benzo[g,h,i]perylene (D12) is used as internal standard.

### 3.5. Quantification and Quality Assurance

The method was validated for recovery, sensitivity, specificity, repeatability and reproducibility, in order to fulfill the performance criteria for methods of analysis of the PAH4 set by Regulation 836/2011 EC [32]. The analytical method fulfilled the requirements established in the relevant 657/2002 EC Decision concerning the performance criteria and other requirements for analytical methods for organic residues and contaminants [44]. The quantification of concentrations and recoveries was carried out by isotopic dilution method, using deuterated PAHs as internal standards. For those native PAHs for which there were no corresponding D-labeled PAHs, the one with the closest retention time (Rt) was used. Recovery rates of labeled internal standards were estimated as average recoveries measured for each analyte in spiked oil samples at concentration levels of 2 ng g^−1^. Relative response factors (RRF) were used to confirm linearity over a five-point calibration range. The LOD was calculated from the lowest concentration with acceptable signal-to-noise ratio, ion abundance ratio within ±15% of the theoretical value and deviation of the relative response factor from the mean value ≤ 20%. Accuracy measurements for PAHs were assessed by the analysis of spiked olive oil samples at two concentration levels (2 and 5 ng g^−1^). Six replicates for each concentration level were analyzed and tested. In every sequence, a blank sample was included in order to check for background contamination, while the reproducibility and repeatability of the method were checked with a quality control chart using spiked olive oil as reference material.

### 3.6. Statistical Analysis

Statistical analysis of data was performed with Origin 2017 SR1. One-way ANOVA tests were applied to determine significant differences among different sampling sites, seasons and molecular weights. *p* values < 0.05 indicate statistically significant difference. Descriptive statistics was used to calculate averages, means and medians of PAH concentrations.

## 4. Conclusions

Farmed mussels originating from the Central Macedonia Region were studied, for the first time to this extent, for complete monitoring of PAH contamination with a highly selective and sensitive method using isotope dilution and GC-MS/MS spectrometry. LODs were in the range of 0.006–0.033 μg kg^−1^ and LOQs between 0.02 and 0.10 μg kg^−1^, while recovery values were from 62 to 119%. Overall, the levels of PAHs in mussels from all sampling sites were found at moderate to low concentrations compared to results from studies in mussels derived from other coastal areas of the Mediterranean Sea. None of the samples exceeded the maximum levels for BaP and sum of four PAHs set by the EU. Of the 26 PAHs studied, 22 were quantified and four found below the LOQ values. LMW PAHs were the predominant PAHs in samples. Spatial variation was not significant, but a statistically significant variation in PAH levels and distribution patterns was observed depending on the season of sampling. It is suggested that continuous screening for the presence of PAHs be performed, as farm units in the Strymonian and Thermaikos Gulfs are important for Greek aquaculture of mussels and other bivalve molluscs.

## Figures and Tables

**Table 1:** Average, standard deviation (SD), relative standard deviation (%RSD), and trueness of the method at two spiked concentration levels 2 and 5 ng g^−1^ for each of the PAH4 of E.C 836/2011.

	Target Value	Average	SD	%RSD	%Trueness
Benz(a)anthracene	2	2.37	0.17	7.23	18.67
	5	5.41	0.29	5.35	8.20
Chrysene	5	2.16	0.08	3.65	8.00
	5	4.97	0.15	3.03	−0.67
Benzo[b]fluoranthene	2	2.19	0.22	9.86	9.61
	5	5.53	0.26	4.79	10.67
Benzo[a]pyrene	2	2.21	0.12	5.52	10.50
	5	5.58	0.13	2.26	11.69
Σ_4PAHs_	8	8.94	0.15	6.57	11.69
	20	21.49	0.21	3.86	7.47

**Table 2:** Sampling locations and number of sampling stations.

Sampling Period	Regional Unit of Thessaloniki	Regional Unit of Imathia and Pieria	Regional Unit of Chalkidiki and Serres
Spring: 21/05/2018–30/05/2018	S1.spring 9 sampling stations	S2.spring 5 sampling stations	S3.spring 3 sampling stations
Winter: 04/02/2019–20/05/2019	S1.winter 9 sampling stations	S2.winter 5 sampling stations	S3.winter 3 sampling stations
Summer: 08/07/2019–09/07/2019	S1.summer 9 sampling stations	S2.summer 5 sampling stations	S3.summer 3 sampling stations

**Table 3:** LOD, LOQ, upper bound mean and median concentrations and range of mean values for each PAH, per site (S1, S2, S3), for the three sampling periods, expressed as μg kg^−1^ w.w.

			S1			S2			S3		
PAH	LOD	LOQ	Mean	Median	Range	Mean	Median	Range	Mean	Median	Range
Na	0.016	0.05	0.10	0.05	<LOQ–0.85	0.08	0.05	<LOQ–0.54	0.24	0.05	<LOQ–1.72
Acl	0.006	0.02	0.31	0.18	<LOQ–2.11	0.20	0.09	<LOQ–1.42	0.19	0.18	<LOQ–0.48
Ac	0.006	0.02	0.07	0.02	<LOQ–0.51	0.06	0.04	<LOQ–0.28	0.11	0.11	<LOQ–0.29
Fl	0.006	0.02	0.82	0.63	0.08–2.99	0.36	0.37	0.08–0.81	0.56	0.58	0.13–0.98
Phe	0.006	0.02	1.78	0.74	0.28–7.62	0.62	0.45	0.28–2.13	0.95	0.53	0.35–2.43
An	0.006	0.02	0.07	0.06	<LOQ–0.29	0.04	0.04	<LOQ–0.07	0.09	0.06	<LOQ–0.46
Fa	0.006	0.02	0.91	0.20	0.06–3.75	0.31	0.16	0.06–1.39	0.94	0.11	0.04–5.07
Py	0.006	0.02	0.27	0.09	0.02–1.24	0.82	0.10	0.02–9.29	0.41	0.13	0.02–2.56
BcFl	0.006	0.02	0.07	0.02	<LOQ–0.45	0.04	0.02	<LOQ–0.16	0.10	0.04	<LOQ–0.47
BaA	0.006	0.02	0.05	0.02	<LOQ–0.24	0.04	0.02	<LOQ–0.23	0.06	0.03	<LOQ–0.24
Chr	0.006	0.02	0.24	0.09	<LOQ–1.44	0.29	0.07	<LOQ–1.57	0.42	0.07	<LOQ–2.01
Cpp	0.006	0.02	0.04	0.02	<LOQ–0.14	0.10	0.03	<LOQ–0.48	0.05	0.02	<LOQ–0.27
5MeChr	0.006	0.02	0.09	0.08	<LOQ–0.54	0.10	0.06	<LOQ–0.46	0.13	0.06	<LOQ–0.76
BbFa	0.006	0.02	0.07	0.05	<LOQ–0.29	0.10	0.05	<LOQ–0.45	0.21	0.05	<LOQ–1.41
BkFa	0.006	0.02	0.06	0.02	<LOQ–0.36	0.06	0.02	<LOQ–0.24	0.25	0.03	<LOQ–1.88
BjFa	0.006	0.02	0.06	0.03	<LOQ–0.32	0.06	0.03	<LOQ–0.23	0.24	0.03	<LOQ–1.69
BaP	0.006	0.02	0.09	0.02	<LOQ–0.47	0.09	0.02	<LOQ–0.34	0.29	0.02	<LOQ–2.11
BeP	0.006	0.02	0.10	0.06	<LOQ–0.40	0.14	0.06	<LOQ–0.66	0.27	0.04	<LOQ–1.87
Per	0.006	0.02	0.22	0.09	<LOQ–1.20	0.22	0.14	<LOQ–0.94	0.14	0.05	<LOQ–0.71
IP	0.006	0.02	0.03	0.02	<LOQ–0.10	0.04	0.02	<LOQ–0.12	0.06	0.02	<LOQ–0.25
DBahA	0.016	0.05	0.05	0.05	<LOQ	0.05	0.05	<LOQ	0.05	0.05	<LOQ
BghiP	0.016	0.05	0.05	0.05	<LOQ	0.05	0.05	<LOQ	0.05	0.05	<LOQ
DBalP	0.033	0.10	0.10	0.10	<LOQ	0.10	0.10	<LOQ	0.10	0.10	<LOQ
DBaeP	0.033	0.10	0.10	0.10	<LOQ	0.10	0.10	<LOQ	0.10	0.10	<LOQ
DBaiP	0.033	0.10	0.10	0.10	<LOQ	0.10	0.10	<LOQ	0.10	0.10	<LOQ
DBahP	0.033	0.10	0.10	0.10	<LOQ	0.08	0.10	<LOQ	0.24	0.10	<LOQ
Σ_26PAHs_			5.96	3.59	1.92–16.03	4.27	5.28	1.37–12.33	6.21	2.82	2.03–25.59
Σ_16PAHs_ (EPA)			4.98	2.96	1.07–15.36	3.21	1.96	0.76–11.18	4.88	2.67	1.25–19.42
Σ_15+1PAHs_ (EU)			1.29	0.94	0.70–3.75	1.42	0.87	0.72–4.16	2.32	0.97	0.71–11.47
Σ_4PAHs_			0.45	0.21	0.08–2.42	0.53	0.16	0.09–2.35	0.98	0.16	0.09–5.77

**Table 4:** Upper bound mean concentrations of BaP and PAH sums per sampling period, total seasonal mean, and range of values of the three sampling sites, expressed as μg kg^−1^ w.w.

		Spring			Winter			Summer		
		Mean	Total Seasonal Mean	Total Range	Mean	Total Seasonal Mean	Total Range	Mean	Total Seasonal Mean	Total Range
**BaP**	S1	0.03	0.02	<LOQ–0.06	0.18	0.28	<LOQ–2.11	0.07	0.08	<LOQ–0.34
	S2	0.02			0.15			0.12		
	S3	0.02			0.83			0.02		
**Σ_26PAHs_**	S1	2.70	2.45	1.37–4.50	11.60	10.12	2.28–25.59	3.59	3.95	1.97–12.33
	S2	1.81			5.71			5.28		
	S3	2.77			13.05			2.82		
**Σ_16PAHs_** **(EPA)**	S1	1.83	1.66	0.76–3.40	10.51	8.70	0.13–19.42	2.61	2.97	1.28–11.18
	S2	1.12			4.44			4.09		
	S3	2.06			10.36			2.21		
**Σ_15+1PAHs_** **(EU)**	S1	0.96	0.89	0.72–2.04	2.01	2.67	0.83–11.47	0.90	0.97	0.70–2.17
	S2	0.78			2.29			1.18		
	S3	0.89			5.24			0.84		
**Σ_4PAHs_**	S1	0.16	0.15	0.08–0.26	0.99	1.34	0.13–5.77	0.20	0.21	0.08–0.74
	S2	0.14			1.16			0.28		
	S3	0.16			2.66			0.13		

## Data Availability

Not applicable.

## Supplementary Materials

The following are available, Table S1: Validation parameters: Recovery (%R), target value, average, standard deviation (SD) and relative standard deviation (%RSD) of the method, using 6 replicates of spiked samples at two concentration levels 2 and 5 ng g^−1^, Table S2: PAHs concentrations measured in proficiency tests samples, assigned concentration and z-scores, Figure S1: Native and D-labeled PAH mass spectra for a blank method sample, Figure S2: Native and D-labeled PAH mass spectra for a proficiency test sample (sun oil) from the EURL-PAH, Table S3: Individual concentrations of PAHs in mussels (μm kg^−1^ wet weight).

## Sample Availability

Samples of the compounds in this article are not available from the authors.
