# Supplementary material for: Monitoring of Polycyclic Aromatic Hydrocarbon Levels in Mussels (Mytilus galloprovincialis) from Aquaculture Farms in Central Macedonia Region, Greece, Using Gas Chromatography–Tandem Mass Spectrometry Method
Source: Molecules. 2021 Sep 30;26(19):5953. doi: 10.3390/molecules26195953 (PMC8512793; doi:10.3390/molecules26195953)
Supplement: Supplementary file 1 [file molecules-26-05953-s001.zip › molecules-1385446-supplementary.pdf]

Table S1. Validation parameters: Recovery (%R), target value, average, standard deviation (SD) and relative standard deviation (%RSD) of the method, using 6 replicates of spiked samples at two concentration levels 2 and 5 ng g^{-1}.

Compound	%R	Target value: 2 ng g^{-1}			Target value: 5 ng g^{-1}		
		Average value	SD	%RSD	Average value	SD	%RSD
Na	119	2.51	0.48	19.00	5.19	0.40	7.66
Acl	105	2.58	0.19	7.38	5.42	0.28	5.16
Ac	92	2.25	0.22	9.78	5.20	0.61	11.75
Fl	104	2.19	0.41	18.69	4.57	0.55	11.94
Phe	116	2.58	0.06	2.29	5.35	0.12	2.34
An	106	2.13	0.06	2.72	4.97	0.17	3.41
Fa	92	2.22	0.20	9.21	5.35	0.16	2.99
Py	89	2.27	0.10	4.50	5.32	0.11	2.11
BcFl	89	2.26	0.13	5.74	5.70	0.20	3.43
BaA	70	2.37	0.17	7.23	5.41	0.29	5.35
Chr	72	2.16	0.08	3.65	4.97	0.15	3.03
Cpp	72	2.34	0.17	7.12	5.67	0.55	9.67
5MeChr	72	2.19	0.16	7.13	4.90	0.19	3.88
BbFa	69	2.19	0.22	9.86	5.53	0.26	4.79
BkFa	63	2.27	0.20	8.61	5.21	0.46	8.74
BjFa	63	2.15	0.48	19.88	5.28	0.52	9.85
BeP	76	2.21	0.12	5.52	5.58	0.13	2.26
BaP	76	2.45	0.30	12.22	5.81	0.24	4.21
Per	62	2.14	0.15	6.83	5.53	0.16	2.88
IP	68	1.92	0.08	4.25	4.58	0.18	3.89
DBahA	65	1.92	0.08	4.10	4.65	0.14	2.98
BghiP	70	2.05	0.03	1.55	4.72	0.12	2.52
DBalP	70	2.40	0.27	11.37	5.44	0.56	10.34
DBaeP	70	2.34	0.45	19.15	5.56	0.27	4.80
DBaiP	70	2.24	0.37	16.57	5.39	0.40	7.35
DBahP	70	2.28	0.30	13.12	5.42	0.39	7.15

Table S2. PAHs concentrations measured in proficiency tests samples, assigned concentration and z-scores.

	BaA			Chr			BbFa			BaP		
	**Measured conc.**	**Assigned conc.**	**z- score**	**Measured conc.**	**Assigned conc.**	**z- score**	**Measured conc.**	**Assigned conc.**	**z- score**	**Measured conc.**	**Assigned conc.**	**z- score**
Olive oil	2.90	3.91	1.26	1.65	2.46	1.59	1.25	1.71	1.24	2.99	2.97	0.03
Olive oil	2.38	2.79	0.71	2.33	2.77	0.77	5.12	5.32	0.19	2.54	2.27	0.56
Sun oil	0.84	1.17	*	1.83	2.19	*	2.07	2.60	*	1.45	1.36	*

*No standard deviation value was given from the organizers, for the calculation of z-score.

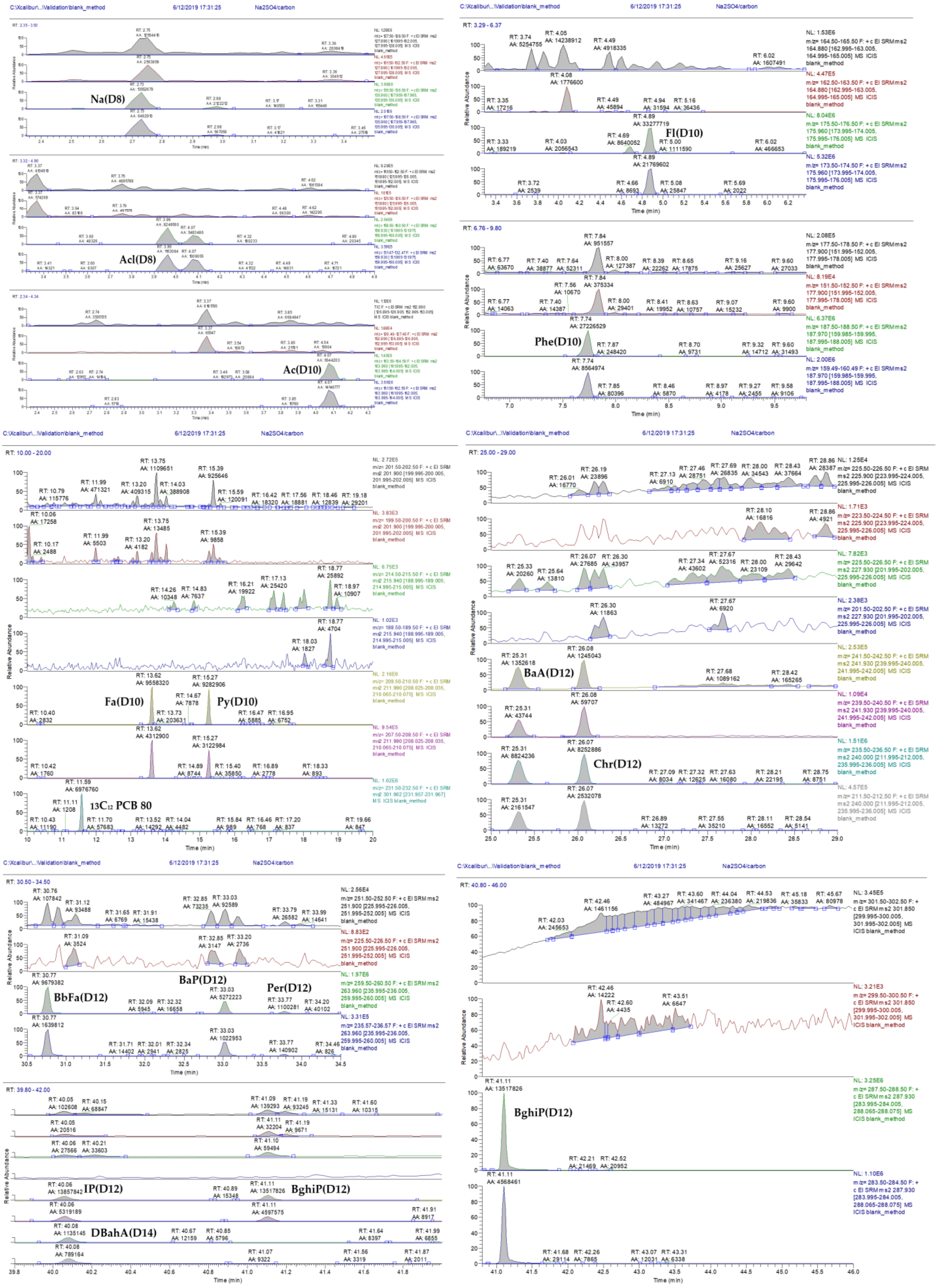

Figure S1. Native and D-labeled PAH mass spectra for a blank method sample.

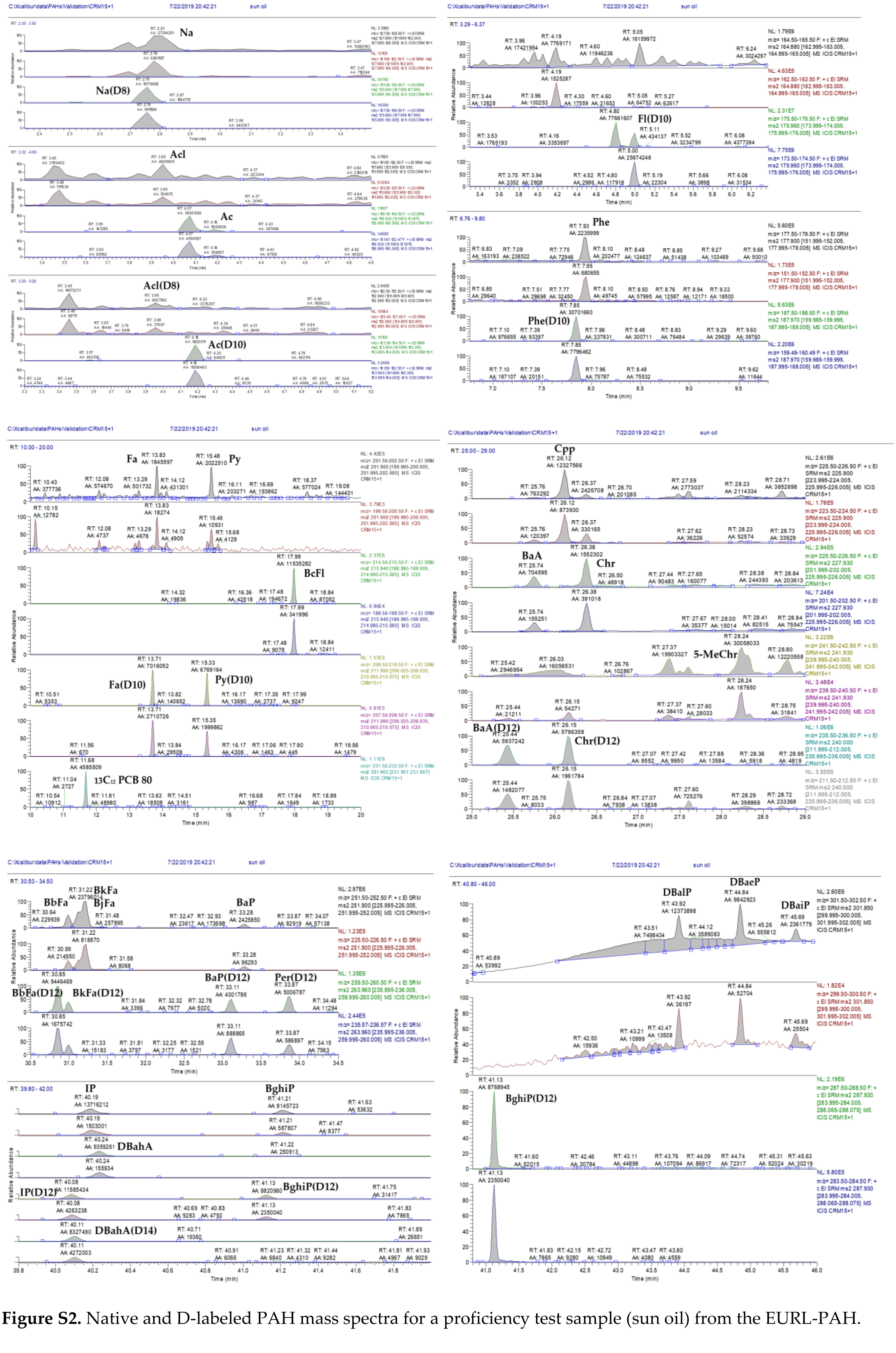

Figure S2. Native and D-labeled PAH mass spectra for a proficiency test sample (sun oil) from the EURL-PAH.

Table S3. Individual concentrations of PAHs in mussels (µm kg^{-1} wet weight)

Sample	PAH (µg kg-1 w.w.)																									
	Na	Acl	Ac	Fl	Phe	An	Fa	Py	BcFl	BaA	Chr	Cpp	5MeChr	BbFa	BkFa	BjFa	BaP	BeP	Per	IP	DBahA	BghiP	DBalP	DBaeP	DBaiP	DBahP
spring																										
S1.a	<0.05	<0.02	0.12	0.27	0.38	0.02	0.23	0.07	0.02	<0.02	0.07	<0.02	0.03	0.06	0.06	0.07	0.06	0.06	<0.02	0.03	<0.05	<0.05	<0.10	<0.10	<0.10	<0.10
S1.b	<0.05	0.09	<0.02	0.93	0.74	0.07	0.18	0.16	0.02	0.03	0.12	0.08	0.12	0.05	0.04	<0.02	0.04	0.31	0.77	0.02	<0.05	<0.05	<0.10	<0.10	<0.10	<0.10
S1.c	<0.05	0.04	0.05	0.08	0.28	0.03	0.18	0.06	<0.02	0.02	0.08	<0.02	0.04	0.04	0.02	0.02	<0.02	0.06	0.29	0.02	<0.05	<0.05	<0.10	<0.10	<0.10	<0.10
S1.d	<0.05	<0.02	0.06	0.12	0.30	0.03	0.20	0.07	0.02	0.02	0.06	<0.02	0.05	0.05	0.02	0.02	<0.02	0.07	0.33	0.02	<0.05	<0.05	<0.10	<0.10	<0.10	<0.10
S1.e	<0.05	0.03	0.04	0.15	0.31	0.02	0.19	0.10	<0.02	0.03	0.09	<0.02	0.04	0.04	0.02	0.02	<0.02	0.06	0.26	0.02	<0.05	<0.05	<0.10	<0.10	<0.10	<0.10
S1.f	<0.05	0.09	0.12	0.28	0.79	0.03	0.31	0.07	<0.02	<0.02	<0.02	<0.02	0.02	<0.02	0.04	0.03	<0.02	<0.02	<0.02	<0.02	<0.05	<0.05	<0.10	<0.10	<0.10	<0.10
S1.g	<0.05	0.05	0.19	0.31	0.80	0.05	0.74	0.22	<0.02	<0.02	0.05	<0.02	0.54	0.17	0.36	0.32	<0.02	0.03	0.02	<0.02	<0.05	<0.05	<0.10	<0.10	<0.10	<0.10
S1.h	<0.05	0.09	0.14	0.30	0.76	0.04	0.27	0.07	<0.02	<0.02	0.03	<0.02	0.02	<0.02	0.03	0.03	<0.02	<0.02	<0.02	<0.02	<0.05	<0.05	<0.10	<0.10	<0.10	<0.10
S1.i	<0.05	0.07	0.12	0.25	0.64	0.03	0.27	0.06	<0.02	<0.02	<0.02	<0.02	0.02	<0.02	0.03	0.03	<0.02	<0.02	<0.02	<0.02	<0.05	<0.05	<0.10	<0.10	<0.10	<0.10
S2.a	<0.05	0.03	0.05	0.15	0.40	0.02	0.19	0.10	<0.02	0.03	0.07	<0.02	0.04	0.04	0.02	0.02	<0.02	0.06	0.20	0.02	<0.05	<0.05	<0.10	<0.10	<0.10	<0.10
S2.b	<0.05	<0.02	0.07	0.15	0.33	0.03	0.19	0.12	0.02	0.02	0.08	<0.02	0.05	0.05	0.04	0.05	<0.02	0.08	0.25	0.02	<0.05	<0.05	<0.10	<0.10	<0.10	<0.10
S2.c	0.54	<0.02	0.04	0.10	0.24	0.02	0.13	0.07	0.02	0.02	0.06	<0.02	0.04	0.04	0.02	0.02	<0.02	0.05	0.09	0.02	<0.05	<0.05	<0.10	<0.10	<0.10	<0.10
S2.d	<0.05	0.05	0.04	0.06	0.20	0.02	0.10	0.05	<0.02	<0.02	0.05	<0.02	0.02	0.03	<0.02	0.02	<0.02	0.04	0.06	0.02	<0.05	<0.05	<0.10	<0.10	<0.10	<0.10
S2.e	<0.05	0.02	0.04	0.07	0.19	<0.02	0.10	0.04	<0.02	<0.02	0.03	<0.02	0.03	0.02	<0.02	<0.02	<0.02	0.03	0.09	<0.02	<0.05	<0.05	<0.10	<0.10	<0.10	<0.10
S3.a	1.72	<0.02	0.17	0.13	0.35	0.02	0.32	0.23	0.04	0.03	0.14	<0.02	0.08	0.05	0.08	0.10	<0.02	0.05	<0.02	0.02	<0.05	<0.05	<0.10	<0.10	<0.10	<0.10
S3.b	<0.05	0.02	<0.02	0.38	0.36	0.02	0.07	0.08	<0.02	<0.02	0.02	<0.02	0.08	0.05	<0.02	<0.02	<0.02	0.04	0.20	<0.02	<0.05	<0.05	<0.10	<0.10	<0.10	<0.10
S3.c	<0.05	0.09	0.29	0.30	0.37	<0.02	0.11	0.02	0.02	<0.02	0.05	<0.02	0.05	0.02	0.03	0.03	0.03	0.03	0.11	<0.02	<0.05	<0.05	<0.10	<0.10	<0.10	<0.10
winter																										
S1.a	0.85	0.48	<0.02	1.43	3.46	0.05	2.05	1.24	0.30	0.22	1.44	0.07	0.11	0.29	0.13	0.14	0.47	0.40	0.16	0.08	<0.05	<0.05	<0.10	<0.10	<0.10	<0.10
S1.b	<0.05	0.33	<0.02	0.90	3.41	0.11	2.66	0.90	0.24	0.24	0.70	0.07	0.10	0.24	0.13	0.12	0.46	0.39	0.38	0.07	<0.05	<0.05	<0.10	<0.10	<0.10	<0.10
S1.c	<0.05	0.23	0.20	0.90	2.74	0.11	2.89	0.67	0.27	0.13	1.15	0.04	0.10	0.14	0.15	0.14	0.22	0.19	0.07	0.08	<0.05	<0.05	<0.10	<0.10	<0.10	<0.10
S1.d	<0.05	0.43	0.13	1.66	3.90	0.13	3.58	0.93	0.45	0.18	1.20	0.04	0.09	0.17	0.06	0.06	0.30	0.26	0.08	0.10	<0.05	<0.05	<0.10	<0.10	<0.10	<0.10
S1.e	<0.05	0.53	0.51	1.35	7.62	0.29	3.75	0.66	0.09	0.04	0.34	0.02	0.06	0.03	<0.02	<0.02	<0.02	0.06	0.02	0.05	<0.05	<0.05	<0.10	<0.10	<0.10	<0.10
S1.f	<0.05	0.76	<0.02	2.07	6.22	0.14	2.91	0.45	0.05	0.06	0.09	0.02	0.09	0.06	0.13	0.14	<0.02	0.05	0.05	0.03	<0.05	<0.05	<0.10	<0.10	<0.10	<0.10
S1.g	<0.05	0.67	<0.02	1.46	6.07	0.17	2.08	0.56	0.03	0.03	0.12	0.04	0.14	0.08	0.06	0.06	0.04	0.12	0.08	0.04	<0.05	<0.05	<0.10	<0.10	<0.10	<0.10
S1.h	<0.05	0.54	<0.02	1.11	3.87	0.09	1.09	0.37	0.04	<0.02	0.08	<0.02	0.08	0.03	0.02	0.03	<0.02	0.04	0.03	0.02	<0.05	<0.05	<0.10	<0.10	<0.10	<0.10
S1.i	<0.05	0.09	<0.02	0.43	0.58	0.05	0.10	0.09	<0.02	<0.02	0.16	0.06	0.14	0.03	0.03	<0.02	0.06	0.05	0.23	<0.02	<0.05	<0.05	<0.10	<0.10	<0.10	<0.10
S2.a	<0.05	0.12	<0.02	0.62	0.58	0.06	0.16	0.07	0.02	<0.02	0.03	0.08	0.07	0.06	0.03	0.04	<0.02	0.04	0.14	<0.02	<0.05	<0.05	<0.10	<0.10	<0.10	<0.10
S2.b	<0.05	0.09	<0.02	0.42	0.45	0.05	0.09	0.06	<0.02	<0.02	0.09	0.04	0.03	0.05	<0.02	<0.02	<0.02	0.05	0.17	<0.02	<0.05	<0.05	<0.10	<0.10	<0.10	<0.10
S2.c	<0.05	0.25	<0.02	0.81	2.13	0.07	1.39	1.24	0.16	0.23	1.57	0.48	0.08	0.45	0.24	0.23	0.10	0.66	0.33	0.12	<0.05	<0.05	<0.10	<0.10	<0.10	<0.10
S2.d	<0.05	0.07	<0.02	0.50	1.03	0.05	0.51	0.36	0.06	0.05	0.92	0.27	0.07	0.22	0.15	0.09	0.32	0.27	0.17	0.07	<0.05	<0.05	<0.10	<0.10	<0.10	<0.10
S2.e	<0.05	0.13	0.14	0.65	1.20	0.04	0.86	0.35	0.12	0.09	1.13	0.02	0.06	0.15	0.22	0.20	0.27	0.24	0.14	0.10	<0.05	<0.05	<0.10	<0.10	<0.10	<0.10
S3.a	<0.05	0.48	0.11	0.78	2.43	0.06	5.07	2.56	0.47	0.24	2.01	0.27	0.76	1.41	1.88	1.69	2.11	1.87	0.71	0.13	<0.05	<0.05	<0.10	<0.10	<0.10	<0.10
S3.b	<0.05	0.27	0.18	0.69	2.39	0.05	1.64	0.45	0.16	0.07	0.72	0.03	0.06	0.09	0.05	0.03	0.16	0.14	0.13	0.06	<0.05	<0.05	<0.10	<0.10	<0.10	<0.10
S3.c	<0.05	0.09	0.13	0.56	1.00	0.07	1.00	0.16	0.09	0.06	0.73	0.03	0.08	0.16	0.11	0.09	0.23	0.20	0.05	0.25	<0.05	<0.05	<0.10	<0.10	<0.10	<0.10
summer																										
S1.a	<0.05	0.35	<0.02	0.49	0.74	0.05	0.14	0.10	0.02	0.02	0.10	<0.02	0.05	0.06	0.02	0.03	0.05	0.04	0.51	0.02	<0.05	<0.05	<0.10	<0.10	<0.10	<0.10
S1.b	<0.05	0.35	<0.02	0.45	0.74	0.08	0.16	0.11	<0.02	<0.02	0.09	0.14	0.15	0.07	<0.02	0.12	0.34	0.29	1.20	<0.02	<0.05	<0.05	<0.10	<0.10	<0.10	<0.10
S1.c	<0.05	2.11	<0.02	0.48	0.66	0.07	0.16	0.09	<0.02	0.03	0.13	<0.02	0.10	0.05	<0.02	0.02	0.07	0.06	0.83	<0.02	<0.05	<0.05	<0.10	<0.10	<0.10	<0.10
S1.d	<0.05	0.10	<0.02	0.69	0.44	0.06	0.09	0.08	<0.02	<0.02	0.03	<0.03	0.07	0.05	0.02	<0.02	0.11	0.09	0.24	0.02	<0.05	<0.05	<0.10	<0.10	<0.10	<0.10
S1.e	<0.05	0.18	<0.02	0.63	0.42	0.06	0.10	0.05	<0.02	0.02	0.13	0.08	0.10	0.03	<0.02	<0.02	<0.02	0.02	0.11	<0.02	<0.05	<0.05	<0.10	<0.10	<0.10	<0.10
S1.f	<0.05	0.14	<0.02	2.99	0.77	0.06	0.08	0.03	<0.02	<0.02	0.02	<0.02	0.11	<0.02	<0.02	<0.02	<0.02	<0.02	0.04	0.04	<0.05	<0.05	<0.10	<0.10	<0.10	<0.10
S1.g	<0.05	0.06	<0.02	0.55	0.43	0.03	0.07	0.02	<0.02	<0.02	<0.02	<0.02	<0.02	<0.02	<0.02	<0.02	<0.02	<0.02	<0.02	<0.02	<0.05	<0.05	<0.10	<0.10	<0.10	<0.10
S1.h	<0.05	0.23	<0.02	1.01	0.59	0.06	0.07	0.03	<0.02	<0.02	0.02	<0.02	<0.02	<0.02	<0.02	<0.02	<0.02	<0.02	0.14	<0.02	<0.05	<0.05	<0.10	<0.10	<0.10	<0.10
S1.i	0.68	0.19	<0.02	0.94	0.51	0.03	0.06	0.03	<0.02	<0.02	<0.02	<0.02	<0.02	<0.02	<0.02	<0.02	<0.02	<0.02	0.09	<0.02	<0.05	<0.05	<0.10	<0.10	<0.10	<0.10
S2.a	<0.05	0.38	0.09	0.27	0.54	0.04	0.25	0.13	0.03	<0.02	0.06	<0.02	0.32	0.04	<0.02	<0.02	<0.02	0.09	0.12	0.03	<0.05	<0.05	<0.10	<0.10	<0.10	<0.10
S2.b	<0.05	1.42	<0.02	0.37	0.39	0.05	0.07	0.06	0.02	0.02	0.02	0.03	0.04	0.04	0.02	0.02	0.02	0.03	0.06	0.02	<0.05	<0.05	<0.10	<0.10	<0.10	<0.10
S2.c	<0.05	0.16	<0.02	0.37	0.32	0.03	0.05	0.03	<0.02	<0.02	<0.02	0.04	0.07	<0.02	<0.02	0.05	0.05	0.04	0.11	<0.02	<0.05	<0.05	<0.10	<0.10	<0.10	<0.10
S2.d	<0.05	0.12	<0.02	0.61	0.48	0.05	0.09	9.29	<0.02	<0.02	0.07	0.08	0.08	0.07	0.04	0.03	0.15	0.13	0.41	0.02	<0.05	<0.05	<0.10	<0.10	<0.10	<0.10
S2.e	<0.05	0.05	0.28	0.30	0.83	0.06	0.46	0.31	0.02	0.02	0.17	0.27	0.46	0.21	0.05	0.10	0.34	0.30	0.94	0.03	<0.05	<0.05	<0.10	<0.10	<0.10	<0.10
S3.a	<0.05	0.23	<0.02	0.58	0.45	0.06	0.11	0.04	<0.02	<0.02	0.05	<0.02	0.06	0.06	<0.02	0.18	<0.02	0.03	0.02	<0.02	<0.05	<0.05	<0.10	<0.10	<0.10	<0.10
S3.b	<0.05	0.18	<0.02	0.98	0.53	0.06	0.04	0.03	<0.02	<0.02	0.03	<0.02	<0.02	<0.02	<0.02	<0.02	<0.02	<0.02	0.03	<0.02	<0.05	<0.05	<0.10	<0.10	<0.10	<0.10
S3.c	<0.05	0.33	<0.02	0.61	0.67	0.46	0.08	0.13	0.07	0.05	0.07	0.02	<0.02	0.02	0.02	<0.02	<0.02	0.03	<0.02	0.04	<0.05	<0.05	<0.10	<0.10	<0.10	<0.10
